# Isolated Depo-Medrol Administration under Tenon’s Capsule for Post-COVID-19 Uveitis in a Child: A Case Report and Literature Review

**DOI:** 10.3390/jcm13051341

**Published:** 2024-02-27

**Authors:** Monika Modrzejewska, Joanna Cyrankiewicz, Oliwia Zdanowska, Wiktoria Bosy-Gąsior

**Affiliations:** 1 Second Chair and Department of Ophthalmology, Pomeranian Medical University in Szczecin, Al. Powstańców Wlkp. 72, 70-111 Szczecin, Poland; 2 2SP ZOZ MSWiA, ul. Jagiellońska 44, 70-382 Szczecin, Poland; 3 Szpital Uniwersytecki im. Karola Marcinkowskiego w Zielonej Górze, 65-046 Zielona Góra, Poland; 4 Scientific Association of Students 2nd Department of Ophthalmology, Pomeranian Medical University in Szczecin, 70-111 Szczecin, Poland

**Keywords:** panuveitis, post-COVID uveitis, steroid therapy, thromboembolic complications

## Abstract

Coronavirus disease 2019 (COVID-19) can manifest with ocular symptoms. These symptoms can be divided into isolated events attributed to COVID-19, and those occurring in multisystem inflammatory syndrome in children (MIS-C), a newly diagnosed disease entity associated with COVID-19 infection. Currently, the literature lacks specific guidelines and treatment regimens for COVID-19 ocular symptoms, especially in children. The authors present the case of a 14-and-a-half-year-old boy with bilateral uveitis of the anterior and posterior segments along with vasculitis and optic neuritis associated with SARS-CoV-2 infection. The authors also perform an up-to-date review of all available publications on the treatment of post-COVID-19 uveitis in children described in the literature between 2020 and 2023. In the case described by the authors, the treatment involved a Depo-Medrol 40 mg/mL injection uder the Tenon capsule, with two subconjunctival injections of epinephrine, topical steroid therapy and non-steroidal anti-inflammatory drugs: dexamethasone 0.1%; diclofenac eye drops. In addition, acetylsalicylic acid (150 mg) and pentoxifylline (100 mg, orally) were administered throughout the course of the disease as well as up to 12 months after its termination, until a complete improvement in visual acuity and the withdrawal of ocular lesions were achieved. It can be assumed that this type of treatment is far more beneficial for pediatric patients, with an effect comparable to systemic steroid administration with a preserved improvement in retinal-vascular circulation, without exposing the child to systemic post-steroid complications.

## 1. Introduction

According to epidemiological data provided by the WHO from 2019, when the first case of SARS-CoV-2 coronavirus infection was described, through 6 December 2023, more than 772 million cases of COVID-19 have been confirmed worldwide. Among them, more than 6.98 million have resulted in death [WHO]. To date, 12 different variants of the SARS-CoV-2 virus have been identified: B.1.1.7 (Alpha), B.1.351 (Beta), B.1.525 (Eta), B.1.427/B.1.429 (Epsilon), B.1.526 (Iota), B.1.617.1 (Kappa), B.1.617.2 (Delta), C.37 (Lambda), P.1 (Gamma), P.2 (Zeta), P.3 (Theta) and B.1.1.529 (Omicron) [1]. In addition to B.1.1.529, there are other Omicron variant lines: BA.2, BA.3, BA.4, BA.5, XBB and BQ.1. On 21 January 2023, it was announced that the XBB.1.5 (Kraken) subvariant had been identified as the cause of 49.1% of COVID-19 cases in the US, but it did not dominate for long, as new Omicron subvariants have emerged since January 2023 [2]. As of 19 August 2023, the most common SARS-CoV-2 Omicron virus variants include: EG.5 (Eris—20.6% of cases); FL.1.5.1 (Fornax—13.3% of cases); and XBB.1.16 (Arcturus—10.7% of cases) [3]. To date, variants of the virus that cause ocular symptoms have been described in the literature, but the severity of clinical presentations between specific viral variants in children remains poorly understood. To date, most analyses of the clinical complications of SARS-CoV-2 variants have been conducted on adults or without a direct link between a specific viral variant and a patient’s clinical phenotype [4]. A study described in 2022 in the US showed a high affinity of the Beta variant for receptors found on the surface of the eye, and an Omicron strain that more often manifests as ocular pruritus than conjunctival congestion [5]. Children may be at risk of severe infection, and a Kawasaki-like disease associated with the clinical manifestations of SARS-CoV-2 has been described in the literature, referred to as the new multisystem inflammatory syndrome in children (MIS-C) [6]. There are reports that during the period of the Delta and Omicron virus infections, patients with MIS-C were younger compared to those when the Alpha variant predominated, which may be related to differences in the pathogenicity of the variants [4]. The virus is transmitted by the droplet route [7], but the genetic material of the virus is also detectable within the eye in tears and conjunctival secretions [7,8,9,10]. Ocular manifestations of COVID-19 infection in children are variable, and the prevalence ranges from 0.7% to 31.6% in children worldwide [11,12,13,14]. They may resolve spontaneously or be the first signs of a severe infection, especially in MIS-C, where accelerated treatment may improve the prognosis [15]. Ocular manifestations so far reported and documented in the course of COVID-19 in children include conjunctivitis, chorioretinitis and/or retinal vasculitis, epitheliitis, corneal epitheliopathy, orbital cellulitis, orbital inflammatory disease, lacrimal gland inflammation, retinal vascular obstruction, retinopathy, maculopathy, intraocular inflammation, cranial nerve palsy and optic neuritis [6,11,16]. It should be noted that in all of the aforementioned cases of ocular infections, treatment is dominated by the use of topical steroids in drops and/or their systemic administration. After analyzing the available literature from 2020 to 2023 with the PubMed and Google Scholar search engines, it was observed that the authors’ description of COVID-19 panuveitis in a child is the first in which a beneficial effect of steroid therapy by injection under the Tenon capsule, with local drip therapy and general multi-month therapy with acetylsalicylic acid and pentoxifylline and without the need for systemic steroid therapy, was demonstrated.

## 2. Materials and Methods

The authors reviewed the current literature covering the years 2020–2023 in the PubMed and Google Scholar search engines, using the following keywords in various combinations: COVID-19, SARS-CoV-2, COVID-19 ocular manifestations, pentoxifylline, COVID-19 thrombotic complications, pediatric ocular cases of COVID-19, panuveitis, treatment of COVID uveitis, Depomedrol under Tenon’s pouch in the treatment of COVID uveitis, ophthalmic complications after COVID infection, post-steroid complications. In addition, references cited in the identified articles were reviewed to find additional reports. This paper describes a post-COVID-19 case of pediatric panuveitis, in the treatment of which, for the first time, the beneficial effect of steroid therapy by injection under the Tenon capsule was demonstrated with local drip therapy and general multi-month therapy with acetylsalicylic acid and pentoxifylline, without the need for systemic steroid therapy. The paper analyzes other cases of pediatric panuveitis described in the literature as well as the latest information on the ocular manifestations of SARS-CoV-2 infection in children.

## 3. Results

### Case Description

**Figure 1:**
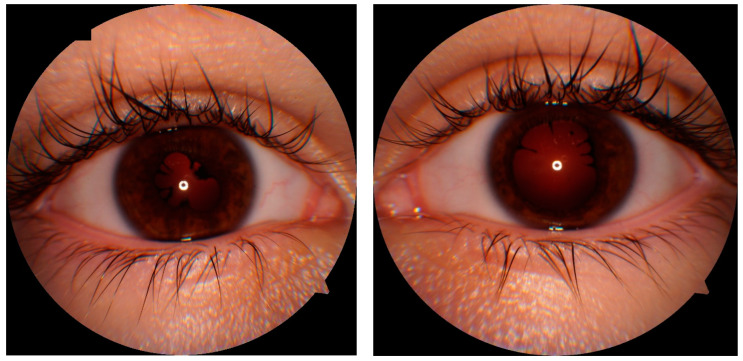
Anterior segment of the right and left eyes (RE and LE). Irregular pupils and posterior synechiae, shallow anterior chamber in the right eye.

**Figure 2:**
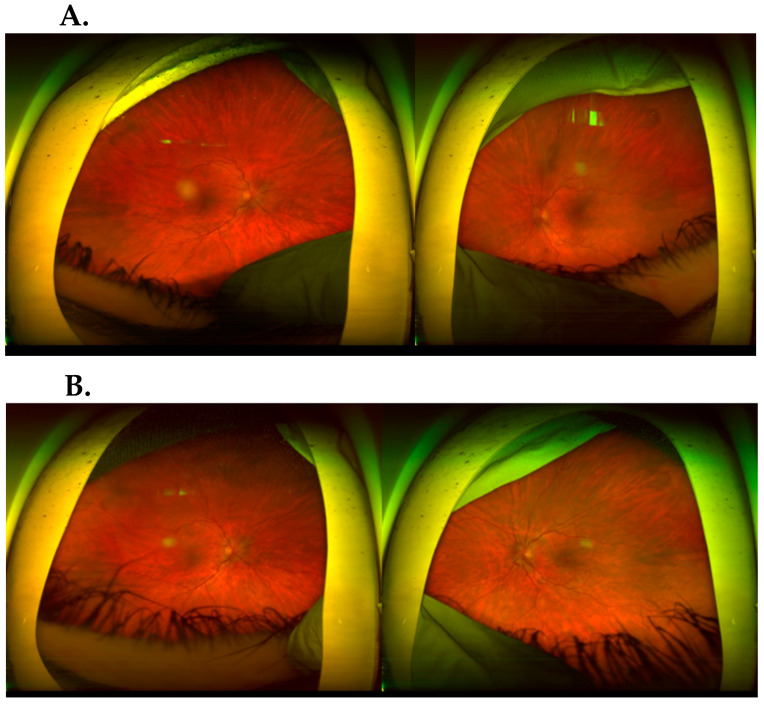
(**A**). Before treatment: fundus of both eyes: optic disc swollen; partially blurred boundaries of the optic disc; hemorrhages on the surface and around the optic disc; tortuous, irregular and dilated retinal vessels; in vitreous posterior vitreous opacification. The fundus of the left eye had signs of intraocular inflammation of the optic nerve disc and retinal vessels. (**B**). Fundus of the right and left eye after treatment.

A 14.5-year-old patient presented to the ophthalmological emergency room with congestion, tearing and markedly increased photophobia of the right eye with symptoms of general infection: malaise, subfebrile state, chills and vomiting. The COVID-19 antigen test performed upon admission was negative. Upon ophthalmologic examination, visual acuity (VA) assessed on a Snellen chart with best spectacle correction was as follows: right eye—0.5 sc, left eye—1.0 sc; binocular intraocular pressure (IOP) 17.6 mmHg (I-care tonometer). Conjunctivitis, of possible COVID-19 etiology, was initially diagnosed in the right eye (RE). Hence, a topical antiviral and antimicrobial treatment was implemented, achieving a mediocre improvement (Viru-POS eye ointment and Vigamox). After 4 weeks, re-exacerbation of ophthalmic symptoms appeared in the left eye, hitherto healthy, resulting in significant visual impairment; VA RE 0.9 sc; VA LE 2.5/50 sc; IOP RE/LE 15.0 and 12.0 mmHg, respectively. The following accompanying ocular symptoms were found under the slit lamp in both eyes: mixed ciliary hyperaemia, corneal edema and multiple inflammatory deposits of the corneal endothelium, tyndalization of the ventricular fluid ++/+++; posterior circular adhesion of the iris, fibrin in the pupillary aperture, constriction and irregular shape of the pupil (Figure 1). In the posterior segment in both eyes there were features of intraocular inflammation of the optic nerve with swelling, obliteration of borders and elevation of the optic disc, streaky hemorrhages on the surface of the disc, retinal vessels mainly venous dilated and tortuous, uneven caliber and constrictions visible in places. An image indicated a prothrombotic condition in the retinal vessels. In the left eye, an image similar to the RE with visible inflammatory vascular sheaths along the retinal vessels in the periocular area was visible (Figure 2A). In the chamber of the vitreous body, hyperechoic exudate was confirmed by ultrasound B. Panuveitis of the left eye and post-uveitis condition of the right eye were diagnosed.

**Figure 3:**
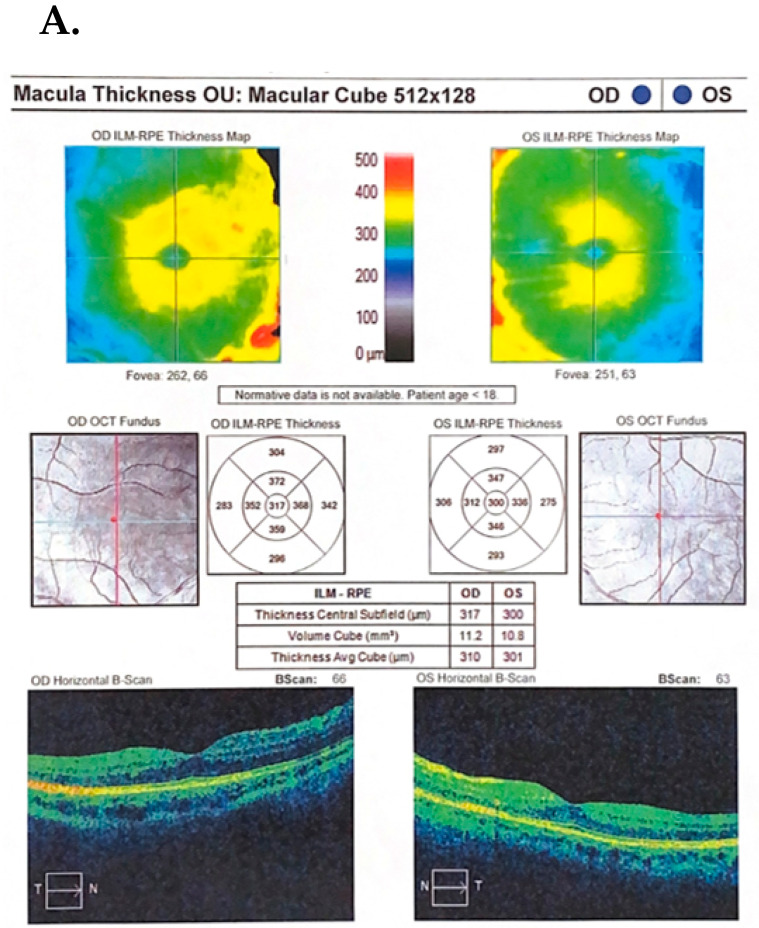

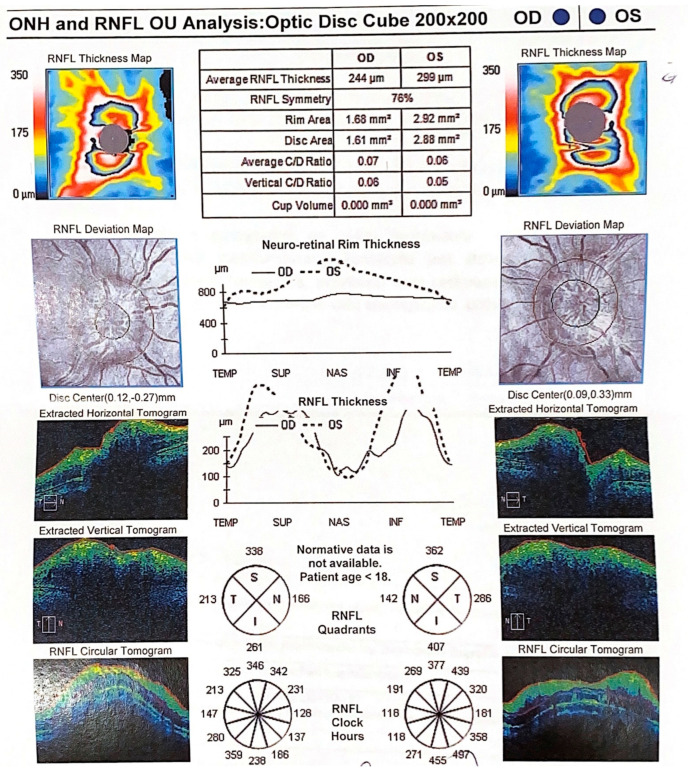

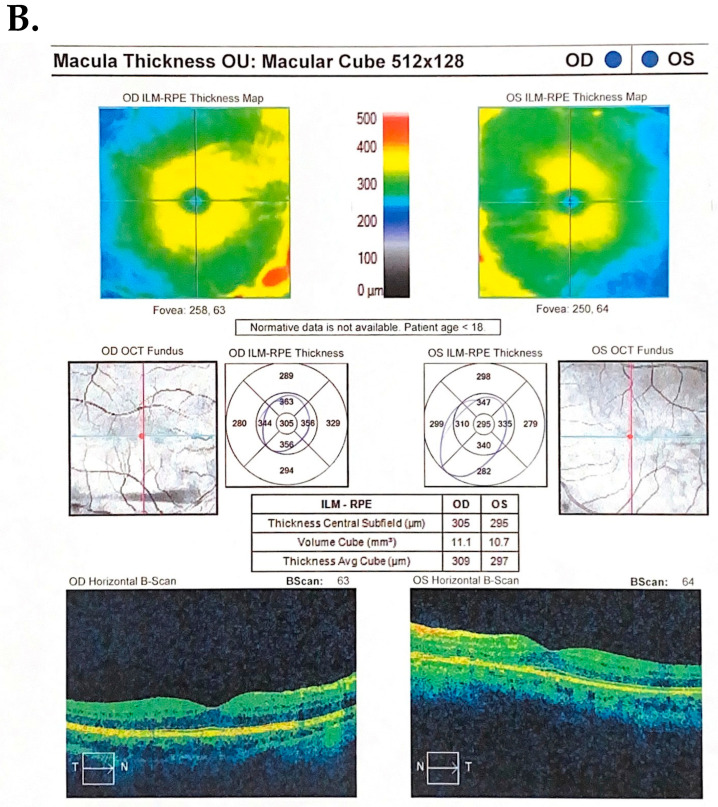

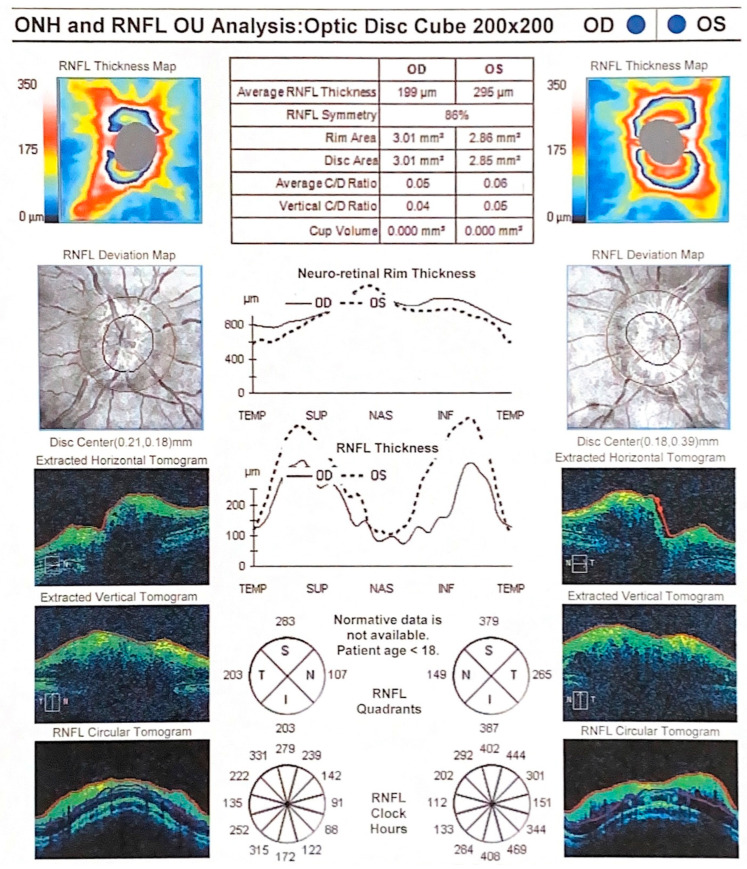
(**A**). Before treatment: on the left side: OCT macular tests for right and left eyes, without lesions. On the right side: OCT optic disc test—significant elevation of the optic nerve; crowding and increased number of the optic nerve fibers. (**B**). After treatment: regression of lesions on the fundus of the eye expressed in the form of improvement in numerical values in OCT examinations: (**A**) macular OCT—right eye 274 μm; left eye 267 μm, (**B**) OCT disc II right/left eye—RNFL right eye 98 μm; left eye 96 μm.

Additional examinations: OCT (optical coherence tomography) of the macula indicated thickening of the central part of the retina in the RE—317 μm, in the LE—300 μm; OCT of the optic disc (CN II)—binocular elevation of the optic nerve disc; crowding of CN II fibers on the disc (more significantly on the left side) with RNFL RE 244 μm, LE 299 μm (Figure 3A). Kinetic perimetry, VER RE/LE and MRI (magnetic resonance imaging) of the brain and orbits within normal limits were visible.

Treatment included a Depo-Medrol 40 mg/mL injection under the Tenon capsule and subconjunctival injection of epinephrine into the RE and LE to disrupt posterior adhesions.

**Figure 4:**
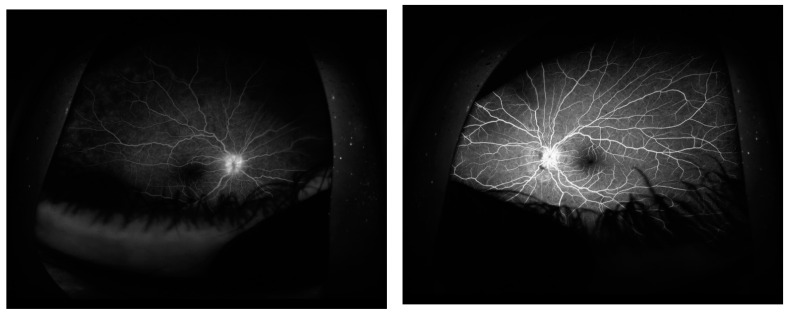
Fluorescein angiography confirmed: blurring of the optic disc boundaries with a dilated cappilares network, fluorescence blocked by the accumulation of dye in the projection of hemorrhages near the LE optic disc, and dilated and tortuous retinal vessels in both eyes. Retinochoroidal leaks were visible in the peripheral retina, which is a symptom of damage to the blood-retinal barrier.

Fluorescein angiography (AF) confirmed hyperfluorescence at the edges of the optic disc with a dilated network of neovascular retinal vessels. Blocked fluorescence in the projection of hemorrhages on the optic disc in LE and dilatation and excessive tortuosity of the retinal vessels in both eyes. Visible leakage of fluorescein at the edge of the optic nerve atrium was a sign of leakage of the blood-retinal barrier (Figure 4).

**Figure 5:**
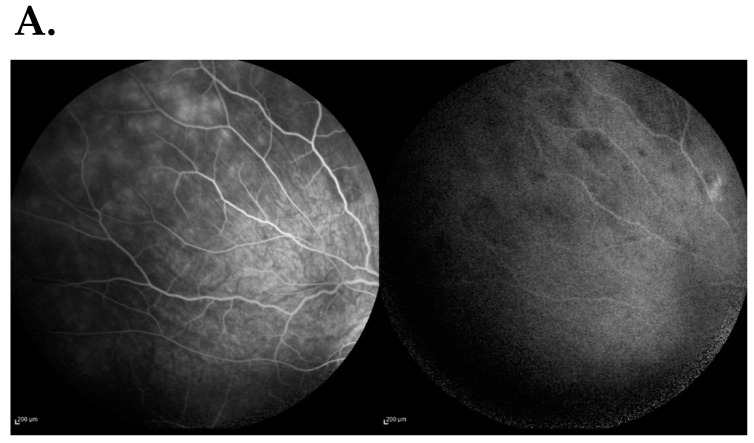

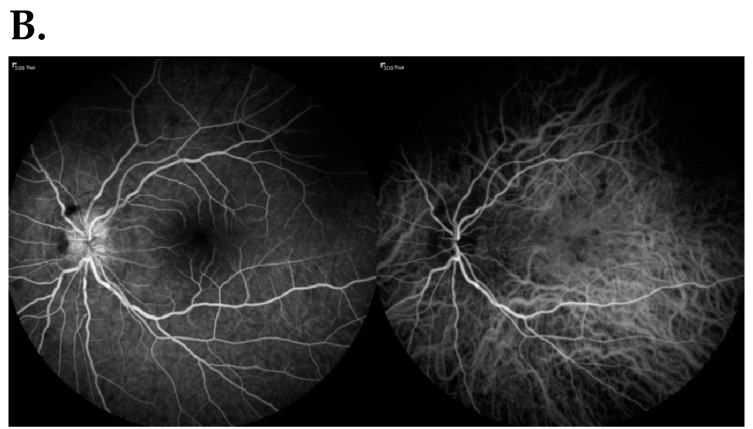
Indocyanine angiography: (**A**) Right eye: there were scattered foci of hypofluorescence in the late phase of contrast in the peripheral part of the uvea. (**B**) Left eye: there were scattered foci of hypofluorescence in the late phase of contrast in the peripheral part of the uvea and in the macular region. These symptoms may correspond to the presence of inflammatory foci in the course of uveitis (**A**,**B**).

Indocyanine angiography:. Late-phase scattered foci of hypofluorescence in uvea’s both eyes were noted. In This was present in the peripheral part of the uvea and in the LE macula, possibly corresponding to the presence of inflammatory foci in the course of uveitis (Figure 5).

Abnormal biochemical test results included: elevated levels of IgG antibodies to SARS-CoV-2 (161 AU/mL; norm less than 12 AU/mL); D-Dimers (501 ng/mL; norm 0–500 ng/mL); and platelets (415,000/mm^3^; norm 150–400,000/mm^3^). IgM p/SARS-CoV-2 antibody levels were within normal limits. Computerized tomography (CT) of the lungs—a ground glass opacity image—and multinodular inflammatory lesions in various segments of the lungs were described, indicating a history of COVID-19 infection. In the multispecialty consultations performed, the following were excluded: JIA (rheumatologist), lymphoproliferative diseases (hematologist), multiple sclerosis (neurologist), upper respiratory tract infections and sinusitis (otolaryngologist), inflammatory foci in the oral cavity (dentist), hypertension and renal failure (hypertensiologist and nephrologist) and tuberculosis and sarcoidosis (pulmonologist).

A detailed laboratory diagnosis of uveitis did not confirm infection for Lyme disease, bartonellosis, toxocariasis, HSV-1, HSV-2, toxoplasmosis, syphilis, cytomegalovirus, leptospirosis, VZV, brucellosis, HIV 1/2, antinuclear antibodies (ANA), anti-neutrophil cytoplasmic antibodies (ANCA), rheumatoid factor or fecal parasites. ACE (angiotensin-converting enzyme) and lysozyme levels were also determined, ruling out sarcoidosis, and HLA-B (human leukocyte antigen) was determined, confirming the presence of HLA B*07 and HLA B*15. 

**Figure 6:**
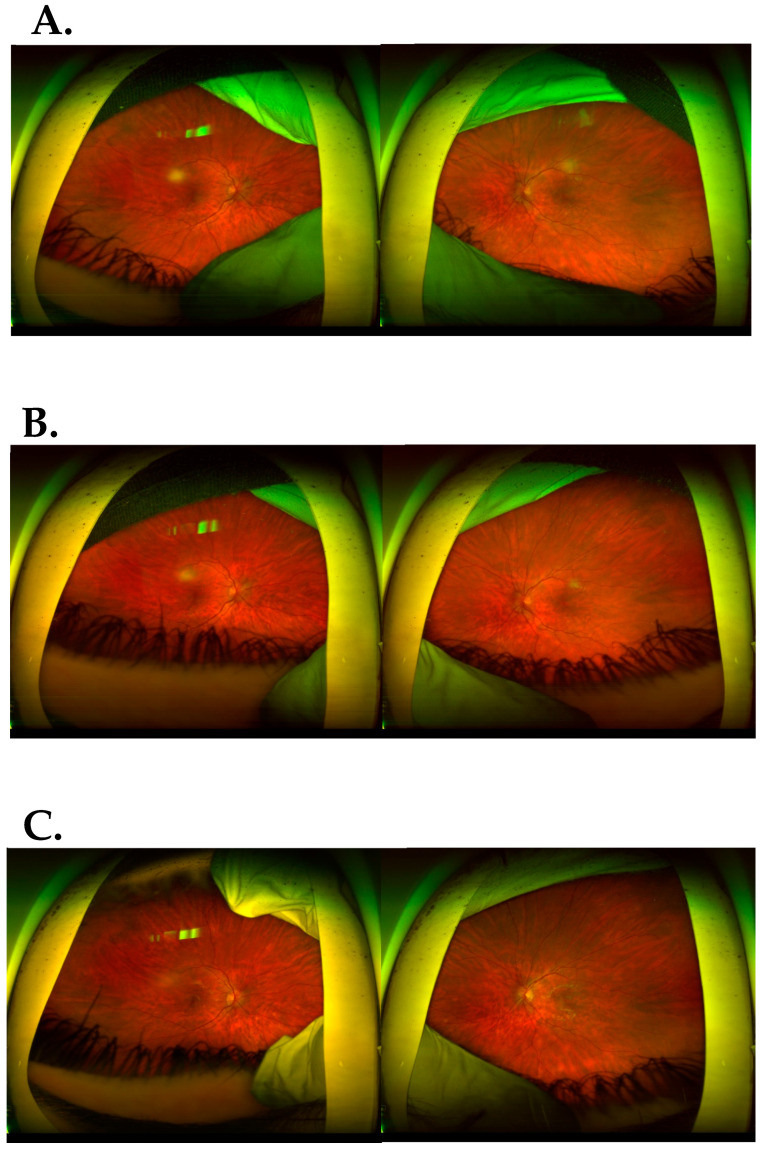
Fundus of the right and left eyes. Evolution of changes on the fundus of the eye (**A**), the fundus of the right eye (RE), the optic disc with already visible borders from the nose, the border of the optic disc from the temple is still blurred, the retinal vessels are still tortuous, still fragmentary, well filled. White spot in the vitreous—much smaller compared to previous tests, less saturated, less dense. Fundus of the left eye: optic disc with borders traceable from the nose, retinal vessels still tortuous in sections, properly filled. The focus in the vitreous body was not visible (**B**). Right and left eyes: evolution of changes at the bottom of the eye (**C**).

Treatment was administered in the form of steroidal and non-steroidal anti-inflammatory eye drops: dexamethasone 0.1%; diclofenac; mydriatic-atropine 1%. Acetylsalicylic acid (150 mg) and pentoxifylline (100 mg) were administered orally, which resulted in a reduction in photophobia and a significant improvement in visual acuity—VRE 1.0; VLE 0.9—as well as a binocular partial disruption of posterior adhesions. Follow-up systematic ophthalmologic examinations conducted initially every 2 weeks for about six months, then every 4 weeks and then every 2 months for up to 1 year after the end of hospitalization, indicated a reduction in the features of intraocular inflammation of the optic disc, absorption of hemorrhages, upright course of retinal vessels, absence of choroidal sheaths and reduction of exudate in the vitreous (Figure 2B and Figure 6).

Diagnostic ophthalmologic examinations after topical steroid and Tenon capsule treatment indicated an improvement in inflammation: in OCT of the macula, a reduction in central retinal thickness (CRT) was obtained in RE 274 μm, and in LE 267 μm. In OCT of the optic disc, a reduction in the thickness of the RNFL was observed in the RE—98 μm and in the LE—96 μm, with swelling of the optic disc area still visible (Figure 3B) On B-scan ultrasound, a reduction in vitreous exudate was noted and laboratory tests showed the normalization of previously elevated or abnormal results.

Topical treatment with Dexamethasone once a day and oral administration of acetylsalicylic acid (150 mg) and pentoxifylline (100 mg) were carried out for up to 12 consecutive months, until complete local improvement was achieved in both eyes.

## 4. Discussion

In a SARS-CoV-2 infection, the angiotensin-converting enzyme 2 (ACE2) is involved in the virus attachment to the host cell. This is a receptor that is a component of the renin-angiotensin system. It is expressed in endothelial cells as well as in corneal and conjunctival cells, and perhaps more internal layers of the ocular epithelium, particularly in fibroblasts and dendritic cells [17]. COVID-19 virus RNA detection in tear samples and conjunctival swabs via polymerase chain reaction (PCR) presents controversial efficacy. A 2020 study by Dawn Ho et al. reported a mere 2.9% positivity rate in eye swabs among 412 samples [18]. Similarly, Nan Hong et al., in their 2020 publication, estimated virus detection in eye swabs to range from 0% to 11% [19]. Another study by Jianhua Xia et al. found that only 0.033% of samples tested positive for COVID-19 virus RNA out of 60 subjects [20]. These findings suggest that while detecting the virus’s genetic material in tears and eye swabs is feasible, the method demonstrates low sensitivity. Expression of the ACE2 gene has been shown to be higher in older children and young adults than in younger children, a likely reason for the lower rate of SARS-CoV-2 transmission through tears in younger children [11]. The ACE2 enzyme is stimulated by the transepithelial serine protease type II TMPRSS2 or cathepsin L. Additional receptors involved in the infection process have also been described thus far: CD147/EMMPRIN/Basigin, Axl and Neuropilin-1 (NRP1) [21]. The conjunctiva, corneal and stroma epithelium as well as the corneal endothelium all show the expression of SARS-CoV-2 receptors such as ACE2, TMPRSS2, CD147 and NRP1 [22]. The conjunctiva is, therefore, the most commonly infected inflammatory structure of the eye, and according to the literature, conjunctivitis occurs in up to 31% of severe COVID-19 cases [6]. According to a June 2021 meta-analysis where a total of 30 studies were identified, the most common ocular symptoms in children and adolescents were conjunctival congestion (7.6%), discharge in the eye (4.8%), tearing (6.9%) and foreign body sensation (6.9%) [11,23]. In January 2021, the statistics compiled by Nasiri et al. were published, according to which the most common ocular symptoms included conjunctivitis, dry eye or foreign body sensation, redness, tearing and itching among 88.8%, 16%, 13.3%, 12.8% and 12.6% of COVID-19-infected patients respectively, regardless of age and gender [24].

Ocular manifestations of coronavirus in pediatric age groups are divided in the literature into two categories: symptoms associated with MIS-C, which include conjunctivitis and anterior uveitis, optic neuritis, retinitis and corneal epitheliopathy; and symptoms associated with COVID-19 such as conjunctivitis, neonatal conjunctivitis (ophthalmia neonatorum), optic neuritis, paralysis of the retrobulbar nerve, oculomotor nerve, opsoclonus, neuromyelitis optica, orbital inflammatory disease (orbital cellulitis, orbital myositis), retinal vasculitis and central retinal vein obstruction [6,16,25]. In addition, other unusual pediatric ocular manifestations of COVID-19 have been described in the literature, such as a case of widespread peripheral necrotizing retinitis in a 5-year-old child, described in 2021. Aniruddh Soni et al. described the association of SARS-CoV2 virus infection with herpes virus reactivation [26]. In February 2021. Pérez-Chimal et al. produced a report on ophthalmic manifestations associated with SARS-CoV-2 in newborns, where the most common symptoms included periorbital edema (100%), chemosis and hemorrhagic conjunctivitis (73%) and ciliary injection (53%). Cases of infants with corneal edema (40%), posterior segment symptoms, including retinopathy of prematurity (in 20% of infants), retinal exudate similar to “cotton wool spots” (13.3%) and vitreous hemorrhage (6.6%) have also been described [27]. In December 2021, Diwakara et al. published the first report of COVID-19-associated rhinocerebral mucormycosis in children and adolescents with type 1 diabetes during treatment for diabetic ketoacidosis [28]. In the patient case presented, features not typical of uveitis but suggesting a possible link to COVID-19 etiology were observed. These included prothrombotic characteristics in retinal vascularization, such as increased tortuosity, irregularity and dilation of the retinal vessels, alongside hemorrhagic strokes visible at the eye fundus. These findings were documented through fundus photography and fluorescein and indocyanine green angiography.

The literature lacks a uniform treatment regimen for uveitis, including panuveitis caused by COVID-19. According to our Internet search (Pubmed and Google Scholar search engines) conducted up until December 2023, only three (3) pediatric cases of panuveitis associated with coronavirus infection in the pediatric population (7, 9 and 12 years old) between 2021 and 2023 have been described in which treatment was successful, but none of the described cases received isolated treatment with steroid injections under the Tenon capsule. All cases were treated with systemic steroids and methotrexate [29], a cycle of intravenous methylprednisolone (IVMP), followed by oral and topical steroids [29] and dexamethasone intravenously (0.4 mg/kg/day) followed by oral steroids, gradually reducing the dose, until the desired effect was achieved [30].

In addition, up until the time of writing this article, no pediatric case of COVID-19-induced intraocular inflammation has been published with treatment consiting of extraocular steroid therapy combined with prophylaxis of retinal vascular thrombotic complications. Precisely such a treatment was applied successfully in our patient, giving him aspirin and pentoxifylline to improve vascular flow, with prothrombotic readiness observed on the fundus (elevation of D-Dimers and retinal vasculature on the fundus and AF).

The literature reports a correlation of COVID-19 infection with the progression of pro-coagulation mechanisms, leading to severe thrombotic complications. The increased affinity of the SARS-CoV-2 virus for the ACE2 receptor in host cells indirectly affects the release of pro-inflammatory cytokines in the vascular endothelium [31]. The body’s excessive inflammatory response, endothelial dysfunction and platelet activation result in an increased predisposition of patients infected with COVID-19 to thromboembolic events, both in the arterial and venous circulation. Due to the abundant vascularization of the retina, this is a structure of the eye particularly vulnerable to thromboembolic complications [32].

In view of the above, it is vital that ophthalmologists pay special attention to the effects of the SARS-CoV-2 virus, which include the increased coagulation caused by the infection. In 2020, F. Seirafianpour et al. described the potential antiviral properties of pentoxifylline and the benefits of its use under many of the circumstances present in COVID-19, particularly in controlling inflammation and related complications [33]. There are also reports of the efficacy of pentoxifylline treatment in retinal vein thrombosis in patients with sudden loss of vision [34,35]. Pentoxifylline decreases blood viscosity, so it can alleviate the increased blood rheology induced by COVID-19 and the hyperaggregation of red blood cells that is associated with the development of coagulopathy. Therefore, it is reasonable to assess further observations in order to confirm the beneficial effects of treatment with isolated steroid therapy under the Tenon capsule in combination with anti-aggregation treatment, thus improving retinal–vascular circulation without exposing the child to post-steroid complications from general use.

## 5. Conclusions

Reviewing the cases highlights that systemic and topical steroids are commonly employed to treat panuveitis—a condition involving inflammation across all sections of the uvea—during COVID-19. Systemic glucocorticoids, recognized for their broad anti-inflammatory and immunosuppressive properties, serve as a foundational treatment for various uveitis types [36]. Their application spans numerous medical fields, particularly for systemic diseases or binocular inflammation, addressing symptoms and reducing post-inflammatory complications such as posterior adhesions, cataracts, macular edema, optic disc edema and retinal vasculitis. In cases where locally administered steroids might be inadequate due to their limited spread, systemic alternatives are preferred [37].

However, systemic steroid therapy can lead to significant adverse effects, including growth and development delays, psychosis, osteoporosis, cataracts, gastrointestinal issues (e.g., peptic ulcers, candidiasis), dermatological problems (hirsutism, striae, impaired wound healing) and hormonal disturbances (weight gain, adrenal suppression, hypertension, hyperglycemia) [38].

Ocular injections and topical glucocorticosteroid drops present a less burdensome alternative for children, sidestepping many systemic complications. Yet, they may necessitate frequent injections, heightening the risk of issues such as increased intraocular pressure, orbital fatty tissue atrophy or loss and ptosis [39]. While intraocular injections carry a risk of inflammation, retinal detachment or vitreous hemorrhage, these complications are infrequent and largely influenced by the injection technique [37].

In the case described by the authors, the treatment used Depo-Medrol (40 mg/mL) injected under the Tenon capsule with two subconjunctival injections of epinephrine, local steroid therapy and non-steroidal anti-inflammatory drugs (dexamethasone (0.1%) and diclofenac drops). In addition, acetylsalicylic acid (150 mg) and pentoxifylline (100 mg) were administered orally throughout the course of the disease as well as up to 12 months after its termination until a complete improvement in visual acuity and withdrawal of ophthalmic lesions were achieved. It can be assumed that this type of treatment is clearly more beneficial for pediatric patients, with an effect comparable to systemic steroid administration, achieving improvement in retinal-vascular circulation without exposing the child to systemic post-steroid complications. During a rheumatological assessment aimed at ruling out systemic connective tissue diseases, HLA-B genotyping was performed, revealing the presence of HLA B*07 and HLA B*15 alleles. The HLA B*15 allele is known to elevate the risk of Behçet’s disease, a form of systemic vasculitis, which could be an etiological factor for uveitis [40,41]. Additionally, the literature identifies a link between the HLA B*07 allele and various systemic conditions, including systemic lupus erythematosus [42,43], ocular histoplasmosis [44] and multiple sclerosis [45,46], as well as its expression in patients with tubulointerstitial nephritis and uveitis syndrome (TINU) [47]. These findings underscore that such diseases may serve as potential etiologies for uveitis.

The 2022 study by Matyushkin D. et al. identified alleles linked to heightened susceptibility to autoimmune processes post-COVID-19, notably HLA A01:01, A26:01, B39:01 and B15:01 alleles [48], which were also found in our patient. These findings suggest a potential association between the detected alleles in the case under discussion and systemic diseases that lead to uveitis, alongside a correlation with autoimmune complications following COVID-19 infection. Such observations might reflect an increased vulnerability of the child’s immune system to autoimmune repercussions post-COVID-19.

## Data Availability

Data regarding the case report are available as electronically registered medical documents, along with confirmation of the patient’s parents and patient by himself declaration in the form of consent to the anonymous use of medical material at the Pediatric Ophthalmology Department of the Medical University of Warsaw in Szczecin, Poland.

## References

[1] Ghosh N., Nandi S., Saha I. (2022). A review on evolution of emerging SARS-CoV-2 variants based on spike glycoprotein. Int. Immunopharmacol..

[2] Parums D.V. (2023). Editorial: The XBB.1.5 (‘Kraken’) Subvariant of Omicron SARS-CoV-2 and its Rapid Global Spread. Med. Sci. Monit..

[3] Parums D.V. (2023). Editorial: A Rapid Global Increase in COVID-19 is Due to the Emergence of the EG.5 (Eris) Subvariant of Omicron SARS-CoV-2. Med. Sci. Monit..

[4] Sperotto F., Gutiérrez-Sacristán A., Makwana S., Li X., Rofeberg V.N., Cai T., Bourgeois F.T., Omenn G.S., Hanauer D.A., Sáez C. (2023). Clinical phenotypes and outcomes in children with multisystem inflammatory syndrome across SARS-CoV-2 variant eras: A multinational study from the 4CE consortium. EClinicalMedicine.

[5] Mostafavi E., Dubey A.K., Teodori L., Ramakrishna S., Kaushik A. (2022). SARS-CoV-2 Omicron variant: A next phase of the COVID-19 pandemic and a call to arms for system sciences and precision medicine. MedComm.

[6] Alnahdi M.A., Alkharashi M. (2023). Ocular manifestations of COVID-19 in the pediatric age group. Eur. J. Ophthalmol..

[7] Worldmeter COVID-19 Coronavirus Pandemic. https://www.worldometers.info/coronavirus/.

[8] Akbari M., Dourandeesh M. (2022). Update on overview of ocular manifestations of COVID-19. Front. Med..

[9] Hosseini S.M., Abrishami M., Zamani G., Hemmati A., Momtahen S., Hassani M., Omidtabrizi A. (2021). Acute Bilateral Neuroretinitis and Panuveitis in A Patient with Coronavirus Disease 2019: A Case Report. Ocul. Immunol. Inflamm..

[10] Braceros K.K., Asahi M.G., Gallemore R.P. (2021). Visual Snow-Like Symptoms and Posterior Uveitis following COVID-19 Infection. Case Rep. Ophthalmol. Med..

[11] Ichhpujani P., Singh R.B., Dhillon H.K., Kumar S. (2023). Ocular manifestations of COVID-19 in pediatric patients. Ther. Adv. Ophthalmol..

[12] Guo C.X., He L., Yin J.Y., Meng X.G., Tan W., Yang G.P., Bo T., Liu J.P., Lin X.J., Chen X. (2020). Epidemiological and clinical features of pediatric COVID-19. BMC Med..

[13] Valente P., Iarossi G., Federici M., Petroni S., Palma P., Cotugno N., De Ioris M.A., Campana A., Buzzonetti L. (2020). Ocular manifestations and viral shedding in tears of pediatric patients with coronavirus disease 2019: A preliminary report. J. Am. Assoc. Pediatr. Ophthalmol. Strabismus.

[14] Mandal A., Kumari E., Roy A., Bandyopadhyay M. (2021). Ocular manifestations and clinical profile of multisystemic inflammatory syndrome in children during COVID-19 pandemic. Int. J. Res. Med. Sci..

[15] Madani S. (2022). Acute and sub-acute ocular manifestations in pediatric patients with COVID-19: A systematic review. Med. Hypothesis Discov. Innov. Ophthalmol..

[16] Singh S., Garcia G., Shah R., Kramerov A.A., Wright R.E., Spektor T.M., Ljubimov A.V., Arumugaswami V., Kumar A. (2022). SARS-CoV-2 and its beta variant of concern infect human conjunctival epithelial cells and induce differential antiviral innate immune response. Ocul. Surf..

[17] Willcox M.D., Walsh K., Nichols J.J., Morgan P.B., Jones L.W. (2020). The ocular surface, coronaviruses and COVID-19. Clin. Exp. Optom..

[18] Ho D., Low R., Tong L., Gupta V., Veeraraghavan A., Agrawal R. (2020). COVID-19 and the Ocular Surface: A Review of Transmission and Manifestations. Ocul. Immunol. Inflamm..

[19] Hong N., Yu W., Xia J., Shen Y., Yap M., Han W. (2020). Evaluation of ocular symptoms and tropism of SARS-CoV-2 in patients confirmed with COVID-19. Acta Ophthalmol..

[20] Xia J., Tong J., Liu M., Shen Y., Guo D. (2020). Evaluation of coronavirus in tears and conjunctival secretions of patients with SARS-CoV-2 infection. J. Med. Virol..

[21] Kyrou I., Randeva H.S., Spandidos D.A., Karteris E. (2021). Not only ACE2-the quest for additional host cell mediators of SARS-CoV-2 infection: Neuropilin-1 (NRP1) as a novel SARS-CoV-2 host cell entry mediator implicated in COVID-19. Signal Transduct. Target Ther..

[22] Collin J., Queen R., Zerti D., Dorgau B., Georgiou M., Djidrovski I., Hussain R., Coxhead J.M., Joseph A., Rooney P. (2021). Co-expression of SARS-CoV-2 entry genes in the superficial adult human conjunctival, limbal and corneal epithelium suggests an additional route of entry via the ocular surface. Ocul. Surf..

[23] Zhong Y., Wang K., Zhu Y., Lyu D., Yu Y., Li S., Yao K. (2021). Ocular manifestations in COVID-19 patients: A systematic review and meta-analysis. Travel. Med. Infect. Dis..

[24] Eissa M., Abdelrazek N.A., Saady M. (2023). COVID-19 and its relation to the human eye: Transmission, infection, and ocular manifestations. Graefes Arch. Clin. Exp. Ophthalmol..

[25] Hu K., Patel J., Swiston C., Patel B.C. (2022). Ophthalmic Manifestations of Coronavirus (COVID-19). StatPearls.

[26] Soni A., Narayanan R., Tyagi M., Belenje A., Basu S. (2021). Acute Retinal Necrosis as a presenting ophthalmic manifestation in COVID 19 recovered patients. Ocul. Immunol. Inflamm..

[27] Pérez-Chimal L.G., Cuevas G.G., Di-Luciano A., Chamartín P., Amadeo G., Martínez-Castellanos M.A. (2021). Ophthalmic manifestations associated with SARS-CoV-2 in newborn infants: A preliminary report. J. Am. Assoc. Pediatr. Ophthalmol. Strabismus.

[28] Diwakar J., Samaddar A., Konar S.K., Bhat M.D., Manuel E., Veenakumari H.B., Nandeesh B.N., Parveen A., Hajira S.N., Srinivas D. (2021). First report of COVID-19-associated rhino-orbito-cerebral mucormycosis in pediatric patients with type 1 diabetes mellitus. J. Mycol. Med..

[29] Ganesh S.K., Mohanan-Earatt A. (2022). An analysis of the clinical profile of patients with uveitis following COVID-19 infection. Indian. J. Ophthalmol..

[30] Merticariu C.I., Merticariu M., Cobzariu C., Mihai M.M., Dragomir M.S. (2022). Pediatric inflammatory multisystem syndrome induced Panuveitis associated with SARS-CoV-2 infection: What the Ophthalmologists need to know. Rom. J. Ophthalmol..

[31] Yeo S., Kim H., Lee J., Yi J., Chung Y.R. (2023). Retinal vascular occlusions in COVID-19 infection and vaccination: A literature review. Graefes Arch. Clin. Exp. Ophthalmol..

[32] Shiroma H.F., Lima L.H., Shiroma Y.B., Kanadani T.C., Nobrega M.J., Andrade G., de Moraes Filho M.N., Penha F.M. (2022). Retinal vascular occlusion in patients with the COVID-19 virus. Int. J. Retina Vitreous..

[33] Seirafianpour F., Mozafarpoor S., Fattahi N., Sadeghzadeh-Bazargan A., Hanifiha M., Goodarzi A. (2020). Treatment of COVID-19 with pentoxifylline: Could it be a potential adjuvant therapy?. Dermatol. Ther..

[34] De Sanctis M.T., Cesarone M.R., Belcaro G., Incandela L., Steigerwalt R., Nicolaides A.N., Griffin M., Geroulakos G. (2002). Treatment of retinal vein thrombosis with pentoxifylline: A controlled, randomized trial. Angiology.

[35] Mostafa-Hedeab G., Al-Kuraishy H.M., Al-Gareeb A.I., Jeandet P., Saad H.M., Batiha G.E. (2022). A raising dawn of pentoxifylline in management of inflammatory disorders in COVID-19. Inflammopharmacology.

[36] Wagner C., Griesel M., Mikolajewska A., Metzendorf M.I., Fischer A.L., Stegemann M., Spagl M., Nair A.A., Daniel J., Fichtner F. (2022). Systemic corticosteroids for the treatment of COVID-19: Equity-related analyses and update on evidence. Cochrane Database Syst. Rev..

[37] Tempest-Roe S., Joshi L., Dick A.D., Taylor S.R. (2013). Local therapies for inflammatory eye disease in translation: Past, present and future. BMC Ophthalmol..

[38] Shivpuri A., Turtsevich I., Solebo A.L., Compeyrot-Lacassagne S. (2022). Pediatric uveitis: Role of the pediatrician. Front. Pediatr..

[39] Abdulla D., Ali Y., Menezo V., Taylor S.R.J. (2022). The Use of Sustained Release Intravitreal Steroid Implants in Non-Infectious Uveitis Affecting the Posterior Segment of the Eye. Ophthalmol. Ther..

[40] Zhong Z., Su G., Yang P. (2023). Risk factors, clinical features and treatment of Behçet’s disease uveitis. Prog. Retin. Eye Res..

[41] Capittini C., Rebuffi C., Lenti M.V., Di Sabatino A., Tinelli C., Martinetti M., De Silvestri A. (2021). Global Meta-Analysis on the Association between Behcet Syndrome and Polymorphisms from the HLA Class I (A, B, and C) and Class II (DRB1, DQB1, and DPB1) Genes. Dis. Markers.

[42] Kong N.C., Nasruruddin B.A., Murad S., Ong K.J., Sukumaran K.D. (1994). HLA antigens in Malay patients with systemic lupus erythematosus. Lupus.

[43] Rigby R.J., Dawkins R.L., Wetherall J.D., Hawkins B.R. (1978). HLA in systemic lupus erythematosus: Influence on severity. Tissue Antigens.

[44] Scharf Y., Zonis S. (1980). Histocompatibility antigens (HLA) and uveitis. Surv. Ophthalmol..

[45] Morris P.J., Vaughan H., Tait B.D., Mackay I.R. (1977). Histocompatibility antigens (HLA): Associations with immunopathic diseases and with responses to microbial antigens. Aust. N. Z. J. Med..

[46] Bertrams H.J., Kuwert E.K. (1976). Association of histocompatibility haplotype HLA-A3-B7 with multiple sclerosis. J. Immunol..

[47] Matsumoto K., Fukunari K., Ikeda Y., Miyazono M., Kishi T., Matsumoto R., Fukuda M., Uchiumi S., Yoshizaki M., Nonaka Y. (2015). A report of an adult case of tubulointerstitial nephritis and uveitis (TINU) syndrome, with a review of 102 Japanese cases. Am. J. Case Rep..

[48] Matyushkina D., Shokina V., Tikhonova P., Manuvera V., Shirokov D., Kharlampieva D., Lazarev V., Varizhuk A., Vedekhina T., Pavlenko A. (2022). Autoimmune Effect of Antibodies against the SARS-CoV-2 Nucleoprotein. Viruses.

